# Assessing the capacity of the health services research community in Australia and New Zealand

**DOI:** 10.1186/1743-8462-2-4

**Published:** 2005-03-08

**Authors:** Jane Pirkis, Sharon Goldfeld, Stuart Peacock, Sarity Dodson, Marion Haas, Jackie Cumming, Jane Hall, Amohia Boulton

**Affiliations:** 1 Program Evaluation Unit, School of Population Health, The University of Melbourne, Melbourne, Australia; 2 Centre for Community Child Health, Royal Children's Hospital, Melbourne, Australia; 3 Public Health Group, Department of Human Services, Melbourne, Australia; 4 Centre for Health Economics, Monash University, Melbourne, Australia; 5 Department of Psychology, The University of Melbourne, Melbourne, Australia; 6 Centre for Health Economics Research and Evaluation, Sydney, Australia; 7 School of Government, Victoria University of Wellington, Wellington, New Zealand; 8 Health Services Research Centre, School of Government, Victoria University of Wellington, Wellington, New Zealand; 9 Te Pūmanawa Hauora, Massey University, Palmerston North, New Zealand

**Keywords:** Health services research, capacity, resources, funding, outputs, Australia, New Zealand

## Abstract

**Background:**

In order to profile the health services research community in Australia and New Zealand and describe its capacity, a web-based survey was administered to members of the Health Services Research Association of Australia and New Zealand (HSRAANZ) and delegates of the HSRAANZ's Third Health Services Research and Policy Conference.

**Results:**

Responses were received from 191 individuals (68%). The responses of the 165 (86%) who conducted or managed health services research indicated that the health services research community in Australia and New Zealand is characterised by highly qualified professionals who have come to health services research via a range of academic and professional routes (including clinical backgrounds), the majority of whom are women aged between 35 and 54 who have mid- to senior- level appointments. They are primarily employed in universities and, to a lesser extent, government departments and health services. Although most are employed in full time positions, many are only able to devote part of their time to health services research, often juggling this with other professional roles. They rely heavily on external funding, as only half have core funding from their employing institution and around one third have employment contracts of one year or less. Many view issues around building the capacity of the health services research community and addressing funding deficits as crucial if health services research is to be translated into policy and practice. Despite the difficulties they face, most are positive about the support and advice available from peers in their work settings, and many are actively contributing to knowledge through academic and other written outputs.

**Conclusion:**

If health services research is to achieve its potential in Australia and New Zealand, policy-makers and funders must take the concerns of the health services research community seriously, foster its development, and contribute to maximising its capacity through a sustainable approach to funding. There is a clear need for a strategic approach, where the health services research community collaborates with competitive granting bodies and government departments to define and fund a research agenda that balances priority-driven and investigator-driven research and which provides support for training and career development.

## Background

As health care costs rise around the world, and the quest for value for money increases, the role of health services research becomes more and more crucial [1-3]. Health services research is a multidisciplinary field of scientific inquiry into questions about the appropriateness, equity, effectiveness and efficiency of different means of improving the health status of individuals and populations. It is broad in its approach, and considers interventions across the spectrum from health promotion and illness prevention through treatment to rehabilitation, recovery and/or palliation, some of which may involve sectors other than health [4,5].

With such an ambitious remit, there is a clear need for a critical mass of health services researchers, equipped with the appropriate skills to conduct high quality research and transfer the findings of this research to policy-makers, planners and practitioners [6]. Internationally, there have been calls to increase the capacity of the health services research community, with demands for investment in training and support, and adequate funding for the research endeavour itself [6,7].

Australia and New Zealand have followed the lead of countries like the United States and the United Kingdom in this movement. Health services research took longer to 'get off the ground' as a coherent discipline here, but similar demands for high quality interventions delivered at the most reasonable cost have led to a greater reliance on evidence from health services research [8]. With the advent of the Health Services Research Association of Australia and New Zealand (HSRAANZ) in 1999, there is now a growing community of health services researchers in these countries [9,10] – in 2004, the HSRAANZ had 112 individual members and 14 corporate members. However, as is the case elsewhere, health services research has remained largely neglected by key granting bodies, such as the National Health and Medical Research Council (NHMRC, Australia) and the Health Research Council (HRC, New Zealand). With only a few exceptions, these bodies have, at best, subsumed health services research under public health research and, at worst, ignored it altogether. So, for example, although Australian researchers have welcomed new NHMRC program grants for health services research, they have noted that these grants have only been introduced after decades of successive reviews have proposed such initiatives, and that they fall short of the recommendations for broader, strategic changes. New Zealand is one step further behind still, with no such grants available.

As a consequence, most of the work of health services research in Australia and New Zealand is conducted at the behest of health departments, and is rarely basic (adding to the conceptual, theoretical or methodological base of the discipline) or investigator-driven. Again, there are exceptions (e.g., funding of the Primary Health Care Research and Evaluation initiative in Australia), but they are limited in number. Funding tends to be project-based, resulting in many health services researchers being employed on tenuous contracts, which culminate in commissioned reports and do not allow for writing peer-reviewed journal articles. There is limited funding for formal training opportunities, which means that most health services researchers come to the discipline by luck, rather than by design. Consequently the capacity of the health services community is still lacking [8-11].

To date, this fairly bleak picture has been largely based on anecdotal evidence, rather than academic inquiry. With the exception of some attempts to quantify the inputs to and outputs of health services research in these countries [9-11], there has been no scientific attempt to assess the capacity of the health services research community in Australia and New Zealand. Indeed, little work of this kind has been done internationally.

The current paper aims to systematically profile the health services research community in Australia and New Zealand, with a view to assessing its capacity. It reports on a survey, conducted to describe the backgrounds and current arrangements of these health services researchers, and to explore their opinions regarding what needs to change if health services research is to maximise its impact. This descriptive work is crucial in an era where health services research is still trying to make its mark.

## Methods

Potential respondents included resident Australians and New Zealanders who were identified from the current individual membership list of the HSRAANZ and the list of delegates of the HSRAANZ's Third Health Services Research and Policy Conference, held in Melbourne from 16–19 September 2003. This yielded a sampling frame of 282 – 37 (13%) were HSRAANZ members only, 179 (63%) were conference attendees only, and 66 (23%) featured on both lists.

On 2 August 2004, emails were sent to all 282 potential respondents, inviting them to complete a confidential, web-based survey. Reminder emails were sent on 16 August, and follow-up phone calls were made on 24 August.

The survey elicited information on respondents' professional role, employment setting, level of appointment, work environment, security of tenure, sources of funding, and research outputs, as well as demographic details. It also invited them to comment on issues they perceived as important in relation to the ability of health services research to have an impact on policy and practice.

Quantitative data were analysed using SPSS and are presented as frequencies and percentages.

Qualitative data were examined to identify major content and themes, and then individual responses were classified according to these themes. The intention was to present 'typical' responses, while at the same time indicating the range of views presented within a given theme.

## Results

### Response rate and sub-sample of interest

In total, 282 invitations to complete the survey were emailed, and 191 individuals submitted responses (68%). All respondents were asked, 'What is your role in health services research, and what percentage of your time do you spend in that role?' One hundred and sixty five (86%) indicated that they conducted or managed health services research, at least part of the time. The remainder commissioned or utilised health services research, but were not actually involved in carrying it out, and are therefore excluded from the following analyses.

### Demographic details

Of the 165 respondents of interest, 136 respondents (82%) were from Australia, 27 (16%) were from New Zealand, and two (1%) had recently begun working overseas. One hundred and three (62%) were female; 62 (38%) were male. Sixteen (10%) were aged less than 35; 48 (29%) between 35 and 44; 68 (41%) between 45 and 54; 33 (20%) 55 or more.

### Academic qualifications

Respondents were asked to indicate their academic qualifications. Of the 162 who provided this information, 56 (35%) indicated that their original undergraduate degree was in Arts, 43 (27%) in Science (including Applied Science, Behavioural Science and Social Science), 28 (17%) in Medicine, 9 (6%) in Economics, 6 (4%) in Business/Commerce/Management, and 3 (2%) some other discipline. Seventeen (11%) did not specify their original degree.

The majority had postgraduate qualifications: 68 (42%) had attained a Masters degree (most commonly a Master of Public Health or Master of Arts) as their highest qualification; 61 (38%) had been awarded Doctorates.

Respondents' qualifications were examined more closely for evidence of clinical backgrounds (e.g., nursing diplomas etc). At a minimum, 56 (35%) had clinical backgrounds of some sort (e.g., medicine, nursing, social work, psychology, physiotherapy, nutrition, health promotion, exercise and pharmacy). This figure is likely to represent an underestimate, since there may have been other respondents with general degrees (e.g., Bachelors of Applied Science) that led to clinical work.

### Professional roles and involvement in health services research

Table 1 provides a picture of the professional roles of respondents. One hundred and twenty one (64%) saw 'health services research' as within their professional role, but other professional roles were also relatively common: 'health policy analysis/planning' was indicated by 75 respondents (39%); and 'other' by 66 (35%). The latter included teaching/education, administration/management of services other than health services, research and evaluation in other allied areas (e.g., social policy analysis), community service provision/management, service development and various types of consultancy. Less common were 'health services management/administration' and 'health care provision', selected by 33 respondents (17%) and 23 respondents (12%), respectively. Typically, respondents worked part time rather than full time in these roles, often juggling more than one role. The mean number of years spent in each role by respondents was around 10, with the exception of 'health care provision' where the average was much higher at nearly 21 years.

**Table 1:** Professional roles by time fraction and years in role

**Health services research**		**Frequency**	**Percentage**
	Full time	50	42.0
	Part time	69	58.0
	Total	119	100.0
	
		**Mean**	**Standard deviation**
	Years in role	9.7	7.5
**Health policy analysis/planning**		**Frequency**	**Percentage**
	Full time	24	36.4
	Part time	42	63.6
	Total	66	100.0
	
		**Mean**	**Standard deviation**
	Years in role	10.4	6.8

**Health services management/administration**		**Frequency**	**Percentage**
	Full time	8	27.6
	Part time	21	72.4
	Total	29	100.0
	
		**Mean**	**Standard deviation**
	Years in role	10.4	5.9

**Health care provision**		**Frequency**	**Percentage**
	Full time	2	10.5
	Part time	17	89.5
	Total	19	100.0
	
		**Mean**	**Standard deviation**
	Years in role	20.9	8.3

**Other professional role**		**Frequency**	**Percentage**
	Full time	28	47.5
	Part time	31	52.5
	Total	59	100.0
	
		**Mean**	**Standard deviation**
	Years in role	10.7	8.5

### Employment setting and arrangements

Table 2 provides a breakdown of respondents' employment settings. Ninety six respondents (58%) were employed in university settings. Government health departments employed 30 respondents (18%), and health services employed 26 (16%). Employment in private companies and self-employment were less common. With the exception of those who were self-employed, respondents tended to be employed on a full-time basis, and the majority described their level of appointment as mid-career or senior.

**Table 2:** Employment setting by time fraction and level of appointment

**University**		**Frequency**	**Percentage**
	Full time	72	75.0
	Part time	24	25.0
	Total	96	100.0
	
	Junior	14	14.9
	Mid-career	35	37.2
	Senior	45	47.9
	Total	94	100.0
**Government health department**		**Frequency**	**Percentage**
	Full time	25	83.3
	Part time	5	16.7
	Total	30	100.0
	
	Junior	0	0.0
	Mid-career	16	53.3
	Senior	14	46.7
	Total	30	100.0

**Health service**		**Frequency**	**Percentage**
	Full time	15	57.7
	Part time	11	42.3
	Total	26	100.0
	
	Junior	2	8.3
	Mid-career	11	45.8
	Senior	11	45.8
	Total	24	100.0

**Private company**		**Frequency**	**Percentage**
	Full time	7	100.0
	Part time	0	0.0
	Total	7	100.0
	
	Junior	0	0.0
	Mid-career	3	50.0
	Senior	3	50.0
	Total	6	100.0

**Self-employed**		**Frequency**	**Percentage**
	Full time	3	23.1
	Part time	10	76.9
	Total	13	100.0
	
	Junior	0	0.0
	Mid-career	1	9.1
	Senior	10	90.9
	Total	11	100.0

### Security of tenure

Respondents were asked about their security of tenure. Of the 157 who responded to this question, 50 (32%) had employment contracts of greater than five years. However, a similar proportion had far more precarious tenure: 23 (15%) had no contract at all, 7 (5%) had less than a one year contract and 18 (12%) had a one year contract. The remaining 59 (38%) had contracts of between two and five years.

### Individual funding sources

Respondents were asked to indicate their general source(s) of current funding. Eighty one (49%) had core funding from their employing institution, 72 (44%) had project funding from key granting agencies, 51 (30%) had funding from government consultancies, and 29 (18%) had funding from other sources, including other government funding, funding from non-government organisations, private funding, self-funding and scholarships.

### Significant funding bodies

Respondents were asked to list the funding bodies they regarded as the most significant in terms of health services research funding in their country, and 148 (90%) did so (see Table 3). Overwhelmingly, they indicated that competitive funding bodies (e.g., the NHMRC and the Australian Research Council in Australia and the Health Research Council in New Zealand) were the most significant: 90 (61%) listed a competitive funding body as their first choice; 45 (30%) as their second choice; and 29 (20%) as their third choice. Similarly, respondents gave considerable weight to government departments as funders (usually the Commonwealth Department of Health and Ageing or State/Territory Health Departments in Australia or the Ministry of Health in New Zealand): 52 respondents (35%) listed a government department as the most significant; 64 (43%) as the second most significant; and 26 (18%) as the third most significant. Other funding bodies deemed to be significant were universities and institutes, health foundations, non-government organisations, philanthropic trusts, private companies, drug companies, insurers and professional bodies, but each was only listed by a small number of respondents.

**Table 3:** Funding bodies regarded as most significant in terms of health services research funding

	**1^st ^choice**		**2^nd ^choice**		**3^rd ^choice**	
	**Frequency**	**Percentage**	**Frequency**	**Percentage**	**Frequency**	**Percentage**
**Competitive funding bodies**	90	60.8	45	30.4	29	19.6
**Government departments**	52	35.1	64	43.2	26	17.6
**Universities and institutes**	2	1.4	4	2.7	5	3.4
**Health foundations**	1	0.7	6	4.1	7	4.7
**Local services**	1	0.7	1	0.7	1	0.7
**Professional bodies**	1	0.7	1	0.7	0	0.0
**Non-government organisations**	1	0.7	0	0.0	4	2.7
**Other**	0	0.0	2	1.4	2	1.4
**Philanthropic trusts**	0	0.0	2	1.4	2	1.4
**Drug companies**	0	0.0	1	0.7	1	0.7
**Private companies**	0	0.0	1	0.7	1	0.7
**Insurers**	0	0.0	0	0.0	2	1.4

### Work environment

Respondents were asked to consider five statements about their respective work environments. Table 4 shows that the majority agreed with the statements 'I work in a setting where peer support for health services research is available' (81%), 'At work, I am co-located with other health services researchers' (65%) and 'I work in a setting where I can easily seek and receive advice about health services research' (81%). There was less agreement with the statements 'I work with a critical mass of health services researchers' and 'The institution/centre/unit where I work has a clear health services research focus', with 41% and 55% indicating that these statements described their work situations, respectively.

**Table 4:** Work environment

**I work in a setting where peer support for health services research is available**		**Frequency**	**Percentage**
	Yes	130	80.7
	No	23	14.3
	Unsure	8	5.0
	Total	161	100.0
**At work, I am co-located with other health services researchers**		**Frequency**	**Percentage**
	Yes	101	64.7
	No	53	34.0
	Unsure	2	1.3
	Total	156	100.0

**I work in a setting where I can easily seek and receive advice about health services research**		**Frequency**	**Percentage**
	Yes	128	80.5
	No	22	13.8
	Unsure	9	5.7
	Total	159	100.0

**I work with a critical mass of health services researchers**		**Frequency**	**Percentage**
	Yes	64	40.8
	No	82	52.2
	Unsure	11	7.0
	Total	157	100.0

**The institution/centre/unit where I work has a clear health services research focus**		**Frequency**	**Percentage**
	Yes	90	55.2
	No	62	38.0
	Unsure	11	6.7
	Total	163	100.0

### Health services research outputs

The research outputs of the sub-sample are shown in Table 5. One hundred and eight respondents (65%) had produced journal articles, 10 (6%) had published books, 37 (22%) had contributed to book chapters, 80 (48%) had written commissioned reports, and 68 (41%) had showcased their work in conference proceedings. In the case of journal articles, commissioned reports and conference proceedings, the majority had produced at least three and sometimes four or more outputs of this kind; for books and book chapters, the norm was one.

**Table 5:** Health services research outputs

**Peer-reviewed publications in academic journals**		**Frequency**	**Percentage**
	1	26	24.1
	2	20	18.5
	3	12	11.1
	4 or more	50	46.3
	Total	108	100.0
**Books**		**Frequency**	**Percentage**
	1	9	90.0
	2	1	10.0
	3	0	0.0
	4 or more	0	0.0
	Total	10	100.0

**Book chapters**		**Frequency**	**Percentage**
	1	22	59.5
	2	9	24.3
	3	2	5.4
	4 or more	4	10.8
	Total	37	100.0

**Commissioned reports**		**Frequency**	**Percentage**
	1	20	25.0
	2	22	27.5
	3	13	16.3
	4 or more	25	31.3
	Total	80	100.0

**Published conference proceedings**		**Frequency**	**Percentage**
	1	16	23.5
	2	17	25.0
	3	12	17.6
	4 or more	23	33.8
	Total	68	100.0

Respondents were also given the opportunity to indicate other outputs in free text. Additional outputs included conference presentations, website publications, non-peer-reviewed journal articles and letters, other reports (e.g., guidelines, manuals, policy documents, methodology papers, reports to study participants, internal reports, research bulletins) and theses.

### Issues impacting upon health services research

Respondents were asked to respond (in free text) to the question, 'What do you think are the most important issues for health services research if it is to have an impact on health policy and planning in Australia and New Zealand?' Several common themes emerged, including building the capacity of the health services research community, funding, translating research into policy/practice, collaborating with others, and undertaking research in specific content areas. Each of these themes is explored in more detail below.

#### (1) Building the capacity of the health services research community

Many respondents viewed building the capacity of the health services research community as a critical issue, commenting on different aspects that needed to be addressed in order for this to occur.

Several felt that the profile of health services research needed to be raised, in order for it to receive greater recognition among funders and decision-makers. Typical comments emphasised: 'increas [ing] recognition of health services research as a distinct and important research stream', 'increasing the profile as a priority area for research' and 'establish [ing] a more visible, cohesive and research-oriented presence as a disciplinary and professional grouping'. These respondents offered a variety of means of achieving this end, most notably ' [seeking] clarity about the role of health services research' by ' [setting] a coherent national research agenda' with agreed priorities, and 'improving the quality of research performed', both in terms of its 'theoretical foundations' and 'methodological rigour'.

Another common theme was the perceived need to improve the 'critical mass of the health services research community', by such means as improving 'continuity and longevity of research groups', developing 'linked network [s] of health services research centres throughout Australasia', and by offering opportunities for 'peer support (e.g., development of the HSRAANZ)'.

Training and career development opportunities were also seen as issues. Many respondents highlighted a need for 'strengthening research training' through 'funding to support capacity building at all levels from [postgraduate] educational programs to furthering professional development of mid to senior researchers' and 'fellowships provided to facilitate the development of health services research as a viable career'. Beyond training, respondents stressed the need for 'nurturing researchers', calling for 'funding and policies to support proper career pathways for health services researchers', 'a proper career structure and transparent promotion arrangements'. Many respondents indicated that a pre-requisite for strengthening career trajectories for health services researchers was improving security of tenure, commenting that 'people need some job security' and 'insecurity of tenure of staff employed on short-term contracts means that experienced people leave for more secure employment elsewhere'.

#### (2) Funding

Over and above funding for training and career development, outlined above, respondents stressed a broader need for 'more funding for longer periods of time' to support 'rigorous, well-planned research'. They observed a need for both 'infrastructure funding' and project funding, particularly for 'larger, in-depth studies ... [as opposed to] ... a lot of smaller projects that duplicate each other'. They noted that health services research has been paid lip-service, being given 'priority ... on paper but not in practice'.

Problems were noted with securing funding from key granting bodies, where there was a perception that these agencies viewed other areas of health as more significant (e.g., 'it's as important as biomedical research but not recognised as such'). Problems were also noted with seeking funding from government departments, which, according to respondents, often view their role as funding health care, and not the research that underpins it (e.g., 'it needs to be funded as an intrinsic component of health service delivery').

#### (3) Knowledge transfer

Many respondents felt that improving knowledge transfer was crucial, making comments like 'there is presently a lack of translation of research results into practice and policy' and 'research translation issues are a priority'.

They suggested several ways in which the current situation could be improved. A number stressed the need to increase the relevance of research to decision-makers, as exemplified by comments like 'the research needs to be relevant to the needs of service providers and policy makers', ' [it] needs to be applied and relevant to contemporary health policy issues' and ' [we must] focus on providing evidence which can help answer key policy questions'.

Many commented on the need for effective dissemination methods, noting that strategies should include 'timely' and 'appropriately written' reports that are 'useful to service providers and policy-makers'. Some also observed that 'being innovative about dissemination of ... evidence' could be helpful, citing alternative strategies such as 'get [ting] into the public media more, get [ting] into policy debates, be [ing] seen and heard'.

Also common were comments about the need for improved relationships between health services researchers and decision-makers, with calls for 'increased collaboration [between] researchers and end-users' and 'increased partnerships between researchers and policy representatives to increase the relevance of research to policy issues'. There was recognition that this would require strategies to involve decision-makers on individual projects (e.g., 'be inclusive of policy-makers on working groups and steering committees'), but that this alone would have 'little impact'. 'Developing strong ongoing relationships between health service researchers and policy-makers around programs of work' and 'frequent and timely dialogue with decision-makers' were seen as important for longer-term, stronger relationships. Several respondents commented that improved relationships between health services researchers and decision-makers would lead to an improved capacity on the part of the former to understand decision-making processes and an increased ability on the part of the latter to commission, understand and interpret health services research.

#### (4) Collaboration with others

Many respondents were positive about the 'multidisciplinary' nature of health services research, but noted that health services researchers need to collaborate with others in order to maximise their contribution. As well as forging stronger relationships with policy-makers and planners (described above), respondents noted that there was a need to develop collaborations with ' [service] providers' and 'partner agencies', to conduct 'joint research with those at the coal face'.

#### (5) Specific content areas for research

A number of respondents saw opportunities to strengthen health services research and contribute to policy and planning decisions by focusing on specific content areas. Commonly identified areas included: health care financing; health system reform; health workforce; health insurance; primary care; hospital care; outcomes, effectiveness and cost-effectiveness; quality of care; and health inequalities.

## Discussion

### Study limitations

The study had several limitations which must be taken into account in interpreting the above findings. Firstly, and most importantly, the study's sampling frame (defined by membership of the HSRAANZ and/or attendance at the Third Health Services Research Conference) may have excluded some health services researchers, introducing some systematic biases. For example, junior and 'commercial' health services researchers may be less likely to join professional associations and attend conferences, which may have artificially inflated the representation of mid-career and senior researchers, and given undue weight to the views of 'academic' health services researchers, respectively. Similarly, New Zealanders may have been under-represented (given the cost of attending a conference in Melbourne), which would have exaggerated the influence of the Australian respondents. However, analyses of the quantitative data indicated that there were no significant differences between respondents on the basis of country of residence. It is acknowledged that the study findings are drawn from a sub-sample of the health services research community, albeit a significant sub-sample, and therefore caution needs to be exercised in extrapolating the findings to the entire health services research community. Defining the sampling frame to ensure broader representation would have been extremely difficult (e.g., identifying a list of 'commercial' researchers), and beyond the financial constraints of the current study.

Secondly, there is a question about the extent to which respondents were representative of the overall sample. The survey's response rate was relatively high, but it may have been that those who did not respond were individuals who had fewest concerns about the state of health services research in Australia and New Zealand. It was not possible to compare the profiles or respondents and non-respondents, since no demographic data were available on the latter.

Thirdly, the survey faced the same problems as all self-report surveys, including issues such as recall bias and social desirability of responses (e.g., the question on respondents' research outputs required them to estimate the total number of each kind of output in the previous year and some may have had difficulty remembering the exact number, and therefore 'rounded up', particularly if they felt that this brought them closer to some imagined norm).

Finally, the survey did not collect any ethnicity data. This was beyond the scope of the survey, but would have been desirable since some of the situations described in the survey may vary for particular population groups. For example, in New Zealand, the introduction of Māori and Pacific Island Career Development Awards has led to a growth in junior health services researchers from indigenous backgrounds who see the awards as a way of gaining qualifications, skills and expertise while working in an area of benefit to their people.

### Interpretation of findings

These limitations aside, the study has largely confirmed the conjecture of key commentators on the capacity of the health services research community in Australia and New Zealand. This community is characterised by highly qualified professionals who have come to health services research via a range of academic and professional routes (including clinical backgrounds), the majority of whom are women aged between 35 and 54 who have mid- to senior- level appointments. They are primarily employed in universities and, to a lesser extent, government departments and health services. Although most are employed in full time positions, many are only able to devote part of their time to health services research, often juggling this with other professional roles. They rely heavily on external funding, as only half have core funding from their employing institution and around one third have employment contracts of one year or less. Many view issues around building the capacity of the health services research community and addressing funding deficits as crucial if health services research is to be translated into policy and practice. Despite the difficulties they face, most are positive about the support and advice available from peers in their work settings, and many are actively contributing to knowledge through academic and other written outputs.

The insecurity of tenure, competing demands on time, paucity of training opportunities, and the poor definition of career pathways described by survey respondents are significant issues. Indeed, health services researchers are taking a stand, lobbying for funding and support commensurate with the importance of their work [10]. There may be a relatively brief window of opportunity within which change must occur, since the current study provides some evidence that the discipline is top-heavy with mid- and senior-level researchers, and that there are not enough junior researchers coming through the ranks. The mid- and senior-level researchers receive strong peer support from a small but cohesive group of colleagues, and are presumably committed to providing sound evidence upon which health policy and planning decisions can be made. But other career options may begin to look attractive and attainable, particularly given their broad discipline base and their high level of qualifications, jeopardising the discipline's critical mass.

The current study provides evidence of a strongly-perceived need for funding commitment for health services research from competitive granting bodies and government departments. Competitive granting bodies are viewed as the most significant funding sources by health services researchers, despite the fact that, collectively, their policies and processes have favoured bio-medical, clinical and public health research over health services research. Government departments are also seen as important, but also have a relatively poor record of supporting health services research, largely viewing it as outside their bailiwick. There is a clear need for a strategic approach, where the health services research community (through the HSRAANZ) collaborates with competitive granting bodies and government departments to define and fund a research agenda that balances priority-driven and investigator-driven research and which provides support for training and career development.

## Conclusion

Unlike many other forms of health research, health services research has the potential to generate savings in the health system by providing evidence about the best way to deliver health care that achieves optimal outcomes at the most reasonable cost. For this reason alone, it would make sense for policy-makers and funders to take the concerns of the health services research community seriously, foster its development, and contribute to maximising its capacity through a sustainable approach to funding.

## Declaration of competing interests

The author(s) declare that they have no competing interests.

## Authors' contributions

JP, SG and SP conceptualised the study and took the lead on designing the survey, and MH, JC and JH helped to refine the sampling frame and survey design. JP and SD undertook the data management and analysis, with input from all other authors. JP took primary responsibility for drafting the original version of the paper, and all other authors contributed substantially to revised drafts. All authors read and approved the final manuscript.
